# Sarm1 deficiency impairs synaptic function and leads to behavioral deficits, which can be ameliorated by an mGluR allosteric modulator

**DOI:** 10.3389/fncel.2014.00087

**Published:** 2014-04-01

**Authors:** Chia-Wen Lin, Chiung-Ya Chen, Sin-Jhong Cheng, Hsiao-Tang Hu, Yi-Ping Hsueh

**Affiliations:** ^1^Institute of Molecular Biology, Academia SinicaTaipei, Taiwan; ^2^Neuroscience Program in Academia SinicaTaipei, Taiwan; ^3^Graduate Institute of Life Sciences, National Defense Medical CenterTaipei, Taiwan

**Keywords:** autism, CDPPB, innate immunity, long-term potentiation, long-term depression, metabotrophic glutamate receptor, *N*-methyl-D-aspartate receptor

## Abstract

Innate immune responses have been shown to influence brain development and function. Dysregulation of innate immunity is significantly associated with psychiatric disorders such as autism spectrum disorders and schizophrenia, which are well-known neurodevelopmental disorders. Recent studies have revealed that critical players of the innate immune response are expressed in neuronal tissues and regulate neuronal function and activity. For example, Sarm1, a negative regulator that acts downstream of Toll-like receptor (TLR) 3 and 4, is predominantly expressed in neurons. We have previously shown that Sarm1 regulates neuronal morphogenesis and the expression of inflammatory cytokines in the brain, which then affects learning ability, cognitive flexibility, and social interaction. Because impaired neuronal morphogenesis and dysregulation of cytokine expression may disrupt neuronal activity, we investigated whether Sarm1 knockdown affects the synaptic responses of neurons. We here show that reduced Sarm1 expression impairs metabotropic glutamate receptor (mGluR)-dependent long-term depression (LTD) formation but enhances *N*-methyl-D-aspartate receptor (NMDAR)-dependent long-term potentiation production in hippocampal CA1 neurons. The expression levels of post-synaptic proteins, including NR2a, NR1, Shank1 and Shank3, are also altered in Sarm1 knockdown mice, suggesting a role for Sarm1 in the maintenance of synaptic homeostasis. The addition of a positive allosteric modulator of mGluR5, CDPPB, ameliorates the LTD defects in slice recording and the behavioral deficits in social interaction and associative memory. These results suggest an important role for mGluR5 signaling in the function of Sarm1. In conclusion, our study demonstrates a role for Sarm1 in the regulation of synaptic plasticity. Through these mechanisms, Sarm1 knockdown results in the impairment of associative memory and social interactions in mice.

## INTRODUCTION

Sarm1 is an evolutionarily conserved adaptor protein that contains, from the N- to C-terminal region, HEAT/Armadillo motifs, sterile alpha motifs (SAMs) and a Toll/interleukin-1 receptor (Tir) domain. The Tir domain provides Sarm1 with unique function as a negative regulator of the Toll-like receptor (TLR) 3 and 4 signaling pathways through interaction with TIR-domain-containing adapter-inducing interferon-β (TRIF; Carty et al., 2006). Although Sarm1 was originally identified in peripheral immune responses (Carty et al., 2006), Sarm1 is predominantly expressed in the brain by neurons (Kim et al., 2007; Chen et al., 2011; Lin et al., 2014). Sarm1 modulates TNF-α production in the brainstem to restrict viral infection (Szretter et al., 2009). Even in the absence of an immune challenge, knockdown of Sarm1 in mice can alter the expression of inflammatory and antiviral cytokines in the brain (Lin et al., 2014), suggesting a neuron-autonomous effect of Sarm1 on cytokine expression. In addition to innate immunity, our previous studies have revealed that Sarm1 participates in neuronal morphogenesis. Through the ASK1-MKK-JNK pathway, Sarm1 can influence microtubule stability and regulate neuronal polarity, axonal outgrowth and dendritic arborization (Chen et al., 2011). Analysis of transgenic Sarm1 knockdown mice also demonstrates that Sarm1 deficiency reduces dendritic arborization and brain size *in vivo* (Chen et al., 2011).

Aberrant immune responses in the brain and impaired neuronal morphogenesis are both involved in the pathogenesis of neuropsychiatric diseases. Our previous study showed that Sarm1 regulates neuronal morphogenesis (Chen et al., 2011). In addition, IL-1ß and TNF-α, the inflammatory cytokines regulated by Sarm1 (Lin et al., 2014), have been found to regulate synaptic plasticity (Del Rey et al., 2013; Gruber-Schoffnegger et al., 2013). Taking consideration with several other lines of evidence, Sarm1 is likely associated with neurodevelopmental disorders. First, the human Sarm1 gene is located at chromosome 17q11 (17:26,698,987-26,728,065), which is within the autism susceptibility locus 6 (Auts6, OMIM%609378, 17:24,000,000-31,800,000). Second, reduced Sarm1 protein levels in the mid-frontal cortex were reported in patients with autism (Azhagiri et al., 2009; Pardo et al., 2009). Finally, the behavioral analysis of Sarm1 knockdown mice also shows intellectual disability, impaired social behaviors and cognitive inflexibility (Lin and Hsueh, 2014). These behavioral defects resemble the characteristics of patients with autism.

In this study, we further investigate the detailed mechanism associated with the behavioral deficits in transgenic Sarm1 knockdown mice. A series of electrophysiology studies were thus performed to analyze Sarm1 knockdown mice. We found that metabotropic glutamate receptor (mGluR)-dependent long-term depression (LTD) is impaired and that *N*-methyl-D-aspartate receptor (NMDAR)-dependent long-term potentiation (LTP) is enhanced in Sarm1 knockdown mice. Moreover, pharmaceutical treatments that aimed to correct the synaptic plasticity were able to improve the animals’ behavioral defects. Our study suggests that Sarm1 regulates electrophysiological responses through the mGluR pathway, which may be relevant to the function of Sarm1 in cognition.

## MATERIALS AND METHODS

### CALCIUM IMAGING

Hippocampal neurons were cultured on a coverslip at a density of 2 x 10^5^, and they were transfected with a Sarm1 shRNA construct (Sarm1i) or a pSuper.neo+GFP vector control (Chen et al., 2011) at six days *in vitro* (DIV) using calcium phosphate precipitation (Chen and Hsueh, 2012). On DIV9, the neurons were washed with 1 ml of 1x Hank’s balanced salt solution (HBSS; 14025-092, Invitrogen) and then incubated with 2 μM Fura-2, AM (F-14185, Invitrogen) in HBSS for 40 min at 37°C. Before recording calcium imaging, the cells were washed three times with HBSS to remove excess Fura-2, and then, the cells were transferred to the recording chamber. The images were recorded using an Inverted Fluorescent microscope (Axiovert 200, Carl Zeiss) equipped with a 20 × /NA:0.75/WD 0.6 mm objective lens and MetaFlour Analyst softwares for acquisition and analysis. The excitation wavelengths of Fura-2 and Fura2-Ca^2^^+^ complex are 380 nm and 340 nm, respectively. The Ca^2^^+^ signal in soma was monitored. The images were taken every 2 s to measure the emission signals. The 340/380 nm excitation ratio was calculated for the indicated intracellular Ca^2^^+^ concentration. After recording a baseline, 50 μM of glutamate in HBSS was continuously perfused into the recording chamber to trigger excitatory responses. The entire Ca^2^^+^ imaging was separated into the baseline, rising and steady phases. The 340/380 ratio in the last 20 cycles (40 s) was also subtracted from the baseline 340/380 ratio to indicate the synaptic activation upon glutamate treatment.

### GOLGI STAINING AND MORPHOMETRY OF DENDRITIC SPINES

The details for the Golgi staining have been described previously (Chen et al., 2011; Wang et al., 2011). Golgi impregnated hippocampal CA1 neurons were captured using a Zeiss AxioImager-Z1 microscope equipped with an AxioCam HR system (Carl Zeiss), a 63 × /1.4 oil (Plan-APO, Carl Zeiss) objective and AxioVision software (Carl Zeiss). All of the z-stack images from each neuron were projected into a single panel by Auto-Montage Pro software (Synoptics Ltd). The number of spines in a 20 μm segment from three secondary apical dendrites of each CA1 neuron were counted using ImageJ.

### PREPARATION OF HIPPOCAMPAL SLICES FOR ELECTROPHYSIOLOGICAL RECORDING

The recording was performed as described previously (Cheng et al., 2011; Hsu et al., 2011), with some modifications. Male mice that were 8–12 weeks old and postnatal day 25–35 were used for the theta-burst stimulation (TBS)-LTP and *(S)*-3,5-dihydroxyphenylglycine (DHPG)-induced LTD studies, respectively. The mice were decapitated, and their brains were isolated by removing the skull with bone cutting forceps. The brains were then immediately placed in ice-cold artificial cerebrospinal fluid (ACSF) for 3 min to lower the temperature before dissecting out the hippocampus. ACSF is made with the following (in mM): 119 NaCl, 2.5 KCl, 1.3 MgSO_4_, 26.2 NaHCO_3_, 1 NaH_2_PO_4_, 2.5 CaCl_2_, and 11 glucose, pH adjusted to 7.4 and gassing with 95% O_2_/5% CO. Transverse hippocampal slices (450 μm thick) were made by vibrotome (Microslicer^TM^ DTK-1000, DKS). The slices were kept in an interface-type holding chamber with oxygenated ACSF (95% O_2_/5% CO_2_) at room temperature (24–25°C) and allowed to recover for at least 90 min before recording.

### EXTRACELLULAR FIELD POTENTIATION RECORDING

Brain slices were transferred to an immersion-type recording chamber, perfused at 2 ml/min with ACSF containing 100 μM picrotoxin at room temperature. An incision was made between the CA1 and CA3 areas to remove afferent input from CA3. For the extracellular field potential recording, a glass pipette filled with 3 M NaCl was positioned in the CA1 stratum radiatum area to record the field excitatory post-synaptic potential (fEPSP). Bipolar stainless steel stimulating electrodes (FHC, USA) were placed in the striatum radiatum to stimulate the Schaffer collateral. Stable baseline fEPSP activity was recorded for at least 10 min by applying a short-duration current stimulation pulse (~ 40 μs) at a predetermined intensity every 15 s. A TBS protocol, with four pulses at 100 Hz in a burst and 10 bursts in a train, was delivered once for LTP induction. A paired-pulse stimulation (50 ms intervals) at 1 Hz for 20 min (1200 trains) with D, L-AP5 applied during recording was used to measure mGluR5-dependent LTD. For paired-pulse facilitation, the stimulation intervals were in a sequence of 50, 100, 200, 300, 500 and 30 ms, with an average of 20 traces. All of the signals were filtered at 1 kHz using the low-pass Bessel filter that was provided with the amplifier, and the signals were digitized at 10 kHz using a CED micro 1401 interface running Signal software (Cambridge Electronic Design, Cambridge, UK). The initial slopes of the fEPSP were measured for data analysis. The synaptic responses were normalized to the average of the baseline. The slope of the fEPSPs recorded during the last 10 min after a different pulse stimulation was averaged for statistical comparisons. All data are presented as means ± SEMs. Statistical significance was tested by unpaired *t*-test. *P* < 0.05 was considered statistically significant.

### WHOLE-CELL RECORDING FOR THE NMDA/AMPA RATIO

Adult male hippocampal slices were cut perpendicularly to the long axis in thickness of 300 μm for the whole-cell voltage-clamp recordings. The slices were transferred to an immersion-type recording chamber mounted on an upright microscope (BX50WI, Olympus Optical Co., Ltd, Tokyo, Japan) equipped with 40x water-immersion objectives, a Nomarski optic system, an infrared-differential interference-contrast microscopy and a CCD camera (Sony XC-EI50, Japan). The oxygenated ACSF was continuously perfused at 1–2 ml/min. Recorded CA1 pyramidal neurons were selected based on their morphology and location. The patch pipettes pulled from borosilicate glass tubing (1.5 mm outer diameter, 0.86 mm inner diameter; G150F-4, Warner Instruments) had a resistance of 5–8 MΩ when filled with internal solution which consisting of the following (in mM): 131 K-gluconate, 20 KCl, 10 HEPES, 2 EGTA, 8 NaCl, 2 ATP, 0.3 GTP, and 6.7 biocytin, with the pH adjusted to 7.2 by KOH and the osmolarity to 300–305 mOsm. Recordings were made at room temperature (24–25°C) with a patch amplifier (Multiclamp 700 B; Axon Instruments, Union City, CA, USA). For voltage-clamp recordings, the series resistance (Rs) was continuously monitored by applying a voltage pulse of 1 mV and was not compensated for. Data were discarded when the Rs varied by > 20% from its original value during the recording. The membrane capacitance of recorded neurons was 30.44 ± 3.22 pF for WT neurons and 36.06 ± 6.96 pF for Sarm1 knockdown neurons (*n* = 6). The membrane resistance of recorded neurons was 327.89 ± 41.70 Mømega for WT neurons and 353.10 ± 54.44 MΩ for Sarm1 knockdown neurons (*n* = 6). All signals were low-pass filtered at a corner frequency of 1 kHz and digitized at 10 kHz using a Micro 1401 interface (Cambridge Electronic Design). Data were collected using Signal software (Cambridge Electronic Design, Cambridge, UK).

For the NMDAR/AMPAR ratio experiments, AMPAR-mediated and NMDAR-mediated excitatory post-synaptic currents (EPSCs) were analyzed in two steps for each neuron. First, the stable synaptic responses (EPSCs) were obtained by holding a neuron at -70 mV. The amplitude of these responses was primarily generated by the α-Amino-3-hydroxy-5-methyl-4-isoxazolepropionic acid (AMPA) receptor because the bath-applied 10 μM 6-cyano-7-nitroquinoxaline-2,3-dione (CNQX) in ACSF completely blocked the current. Next, the holding potential was changed to +40 mV to measure the synaptic responses mediated by the NMDA receptors, which were later blocked by 50 μM AP5 with CNQX perfusion. Twenty traces at each holding potential were averaged to analyze the current mediated by each specific receptor. For the NMDA/AMPA ratio, AMPA or NMDA EPSCs were measured by the amplitude of the peak current.

### CHEMICALS AND TREATMENT IN THE ELECTROPHYSIOLOGY STUDY

The chemicals used for the ACSF and internal solutions were purchased from Merck and Sigma. DL-APV, CNQX, picrotoxin, DHPG, and CDPPB [3-cyano-*N*-(1,3-diphenyl-1H-pyrazol-5-yl) benzamide] were purchased from Tocris. All of the drugs were prepared in a high-concentration stock that was kept in an -80°C freezer. The chemicals were added to an ACSF bath prior to being applied.

### IMMUNOBLOTTING ANALYSIS

The entire hippocampus was homogenized with a pellet pestle in lysis buffer containing 50 mM Tris (pH 7.4), 320 mM sucrose, 2 mM dithiothreitol, 2 μg/ml leupeptin, 2 μg/ml leupeptin, 2 μg/ml pepstatin-A, 2 μg/ml aprotinin, 1 mM buffer tosylphenylalanylchloromethane, and 2 mM phenylmethylsulfonyl fluoride and was then centrifuged at 800x *g* for 10 min at 4°C. The supernatant was boiled at 55°C in SDS sample buffer and separated by 7.5% or 10% SDS-PAGE. After transfer, the membrane was treated with a blocking solution (2.5% non-fat milk in 0.3% TBST) followed by incubation with primary antibodies at 4°C overnight. The proteins were detected with HRP-conjugated goat secondary antibody and developed with Western Lightning Plus ECL (PerkinElmer). The blot images were developed using ImageQuant^TM^ LAS 4000 (GE Health care Life Sciences, USA) and X-ray film (Fujifim) without modification of contrast or brightness.

### BEHAVIOR ANALYSIS

All of the animal experiments were carried out with the approval of the Academia Sinica Institutional Animal Care and Utilization Committee. Sarm1 knockdown mice were generated in a C57BL/6J background (Chen et al., 2011). The animals used in the behavioral assays were the offspring of transgenic males and wild-type C57BL/6J females to eliminate any maternal effects of Sarm1 knockdown on offspring behavior. Male littermate mice were used in the behavioral assays to avoid variations due to the estrus cycle. All of the procedures for the behavioral analyses have been described previously (Chung et al., 2011; Lin and Hsueh, 2014), with minor modifications. The behavioral testing was performed at 8–16 weeks of age. All of the animals were housed in mixed-genotype groups of 3–5 mice per cage. The animals were acclimatized to the test room for at least 1 week prior to the behavioral assays. A 12 h light/dark cycle (lights off at 20:00) was maintained in the test room. Food and water were accessed *ad libitum*. For the reciprocal social interaction assays, each experimental mouse was independently housed for 1 week before the test. The tests were performed between 14:00 and 19:00, prior to the onset of the dark phase. For the rescue experiments, CDPPB was dissolved in DMSO at a concentration of 14.6 mg/ml (40 mM), and it was further diluted in polyethylene glycol-400 (PEG400, Sigma) at a ratio of 1:8 to increase its solubility. Wild-type or Sarm1 knockdown mice received an intraperitoneal (i.p.) injection of CDPPB at a dose of 10 mg/kg, or they received the same volume of a DMSO-PEG400 mixture as a vehicle control. The i.p. injections were performed six hours before the day 0 training for contextual fear conditioning or reciprocal social interaction to give sufficient recovery time from the illness caused by viscous PEG.

### STATISTICAL ANALYSES

Unpaired *t*-tests were performed to analyze the differences between WT and transgenic (Tg) animals using GraphPad Prism 5.0 (GraphPad Software, La Jolla, CA, USA). Two-way ANOVA was used to analyze **Figure 6**. For all comparisons, a *P* value < 0.05 was considered significant.

## RESULTS

### Sarm1 KNOCKDOWN LEADS TO HYPERSYNAPTIC RESPONSES UPON GLUTAMATE STIMULATION IN HIPPOCAMPAL NEURONS

**FIGURE 1:**
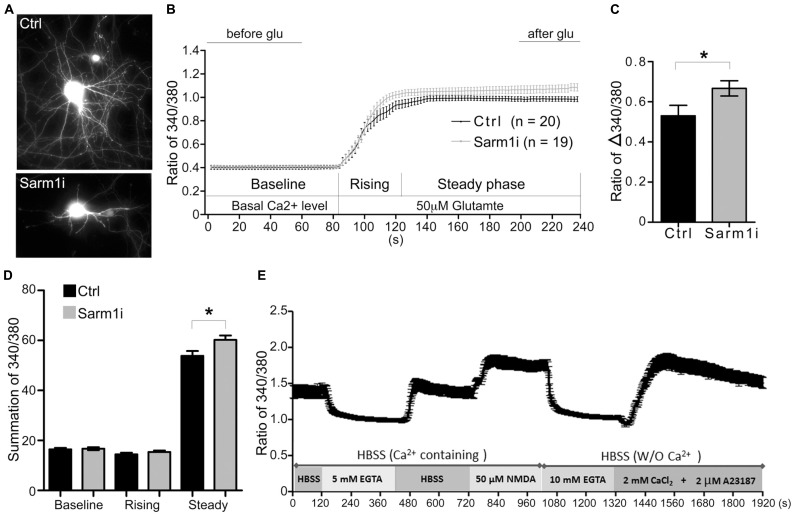
**Sarm1 knockdown enhances Ca^2+^ influx after glutamate treatment in cultured hippocampal neurons. (A)** Representative images of hippocampal neurons that have been transfected with a control vector (Ctrl) or a Sarm1 shRNA expressing construct (Sarm1i). **(B)** Calcium imaging after glutamate stimulation is shown. The Ca^2^^+^ signal in the soma was monitored by Fura-2 for basal Ca^2^^+^ levels between 0 and 90 s. After glutamate perfusion, a strong Ca^2^^+^ influx response was induced, as shown by the dramatic increase in the 340/380 ratio. The entire response was separated into three phases (baseline, rising, and steady), as indicated. **(C)** The ratio of Δ340/380 is shown. The means of the last 40 s (after glu) and the first 60 s (before glu) in **(B)** were calculated to determine the ratio of Δ340/380 upon glutamate stimulation. **(D)** The summations of the 340/380 ratio of the baseline, rising and steady phases shown in **(B)**. **(E)** WT neurons were subjected to a series of treatments, as indicated. After NMDA treatment, the calcium concentration was able to return to the baseline and respond to calcium ionophore A23187 to cause calcium influx. The values represent the means ± SEMs. The sample sizes (*n*) are indicated. *P < 0.05.

To investigate whether Sarm1 knockdown influences synaptic function, Ca^2^^+^ imaging using cultured hippocampal neurons was performed to monitor neuronal activity induced by glutamate treatment. The neurons were transfected with a Sarm1 knockdown construct, Sarm1i (Chen et al., 2011), at DIV 6 and then subjected to Ca^2^^+^ image recording at DIV 9. The Sarm1 knockdown construct co-expresses green fluorescence protein (GFP). Thus, the GFP signal provides a marker for neuronal Sarm1 knockdown and outlines neuronal morphology. We first confirmed that similar to previous findings (Chen et al., 2011), knockdown of Sarm1 impaired dendritic arborization (**Figure 1A**). The Ca^2^^+^ influx induced by glutamate stimulation was investigated in neurons that were transfected with a vector control or Sarm1i at DIV 9 by monitoring intracellular Ca^2^^+^ concentrations, as indicated by Fura-2. After recording the baseline of intracellular calcium concentration, glutamate was added into the culture to induce calcium influx through NMDAR (**Figure 1B**). We found that compared the calcium concentration before and after glutamate stimulation, Sarm1 knockdown obviously induced more calcium influx after glutamate treatment (**Figure 1C**). The entire response was also divided into three phases including baseline, rising and steady phases (**Figure 1B**). For the baseline and rising phases, calcium influx was comparable between control and Sarm1 knockdown neurons (**Figure 1D**). For the steady phase, the calcium concentration of Sarm1 knockdown neurons was noticeably higher than that of control neurons. To make sure that the activation of NMDAR does not induce cytotoxicity during recording, we also monitored whether the calcium concentration is able to return to the baseline after NMDAR activation and further respond to calcium ionophore A23187 (**Figure 1E**). Indeed, the cultures were still healthy after induction of NMDAR opening and were able to return to the baseline and further respond to calcium ionophore A23187 (**Figure 1E**). Taken together, the results of calcium imaging indicated that neurons with Sarm1 knockdown had a higher Ca^2^^+^ influx after glutamate stimulation compared to the vector-transfected control. The enhanced responses to neuronal stimulation suggested that Sarm1 knockdown can change the synaptic composition or the downstream signaling pathway following stimulation.

### Sarm1 KNOCKDOWN INCREASES THE SPINE DENSITY OF CA1 NEURONS AND ENHANCES NMDAR-DEPENDENT SYNAPTIC PLASTICITY IN Sarm1 KNOCKDOWN MICE

**FIGURE 2:**
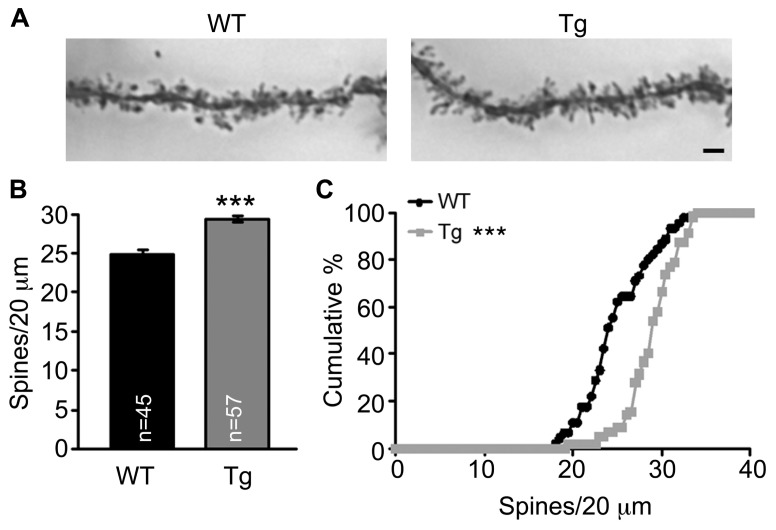
**Sarm1 knockdown increases dendrite spine density in the hippocampal CA1 region. (A)** The representative images of CA1 neuronal Golgi stains are shown. **(B)** The mean ( ± SEM) and **(C)** cumulative probability of the numbers of dendritic spines were compared between WT and Sarm1 knockdown CA1 neurons. The scale bars are set at 5 μm. The sample sizes (*n*) of the dendrite number are indicated in the panels. The data were collected from 15 to 19 neurons from 4 WT to 4 Sarm1 knockdown mice, respectively. ***P < 0.001.

Because spine morphology is linked with and changed by synaptic activity, we then examined whether Sarm1 deficiency alters the morphology and density of dendritic spines of the CA1 neurons. Golgi stain was used to analyze Sarm1 knockdown mice, which were established previously (Chen et al., 2011). The results showed that the dendritic spines of Sarm1 knockdown neurons tended to be elongated in their morphology (**Figure 2A**). However, the length and width of individual dendritic spines were immeasurable because the spines were bunched together. Thus, we only quantified the density of the dendritic spines. The data showed that Sarm1 knockdown resulted in a higher density of dendritic spines in CA1 neurons (**Figures 2B,C**). This increase in dendritic spine density echoes the increase of calcium concentration in Sarm1 knockdown neurons after glutamate stimulation.

**FIGURE 3:**
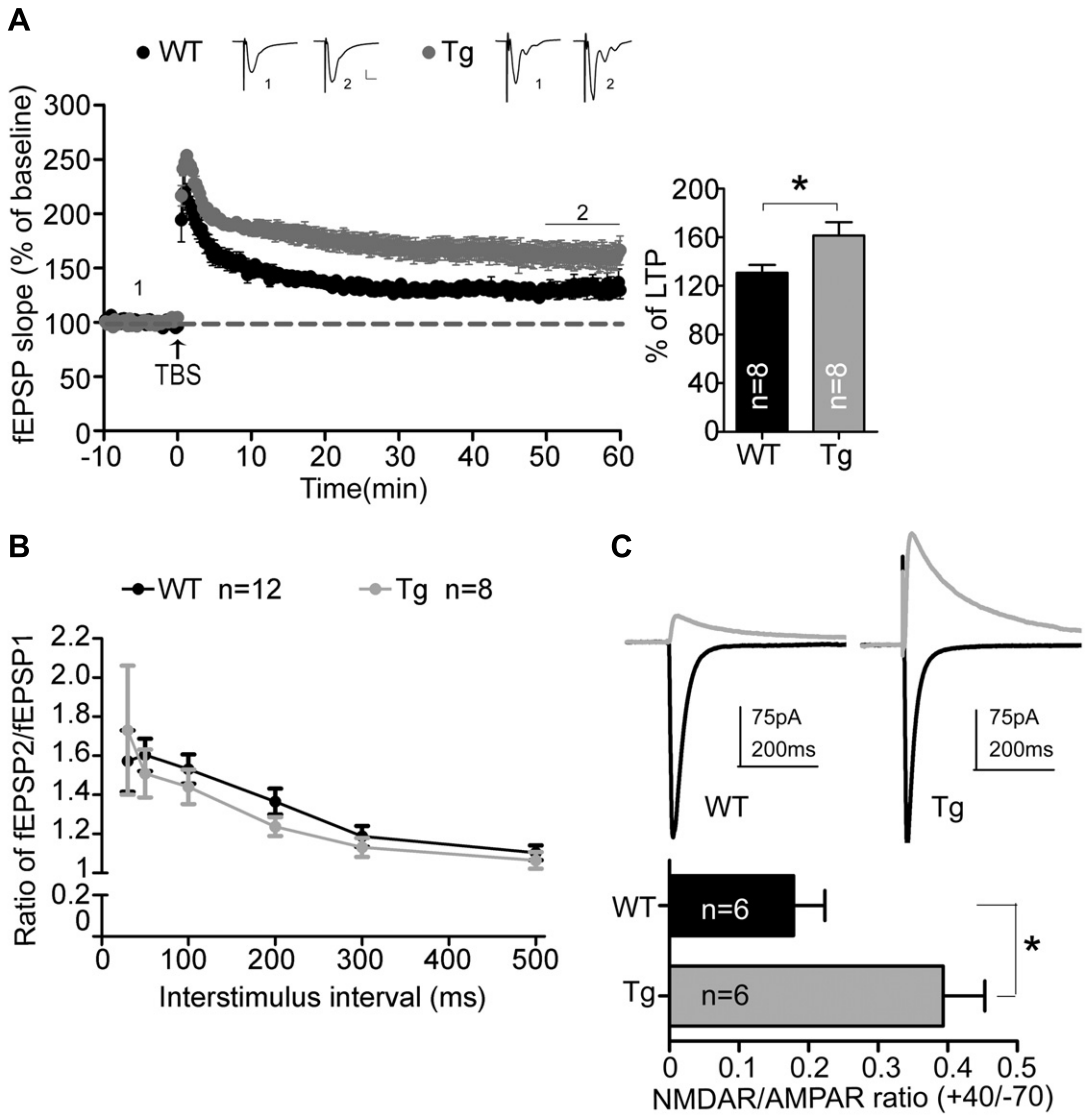
**Sarm1 knockdown enhances NMDAR-dependent LTP in hippocampi. (A)** Theta burst stimulation-induced LTP was recorded in the hippocampal CA1 region. The fEPSPs were recording in the CA1 and plotted against time. The representative traces shown at the top are an average of 40 fEPSPs from the baseline (1) and the last 10 min of recording (2). Each trace is the average of 40 fEPSPs from the baseline. The last 10 min of recording are further subjected for quantitative analysis. The horizontal dashed lines indicate the average value of the normalized amplitude during the baseline period. Calibration: 0.2 mV, 10 msec. The histogram summarizing the average fEPSP of the last 10 min of recording is shown in the right panel. The data represent the mean plus SEM (for histograms) or ± SEM (for fEPSP slope). *P < 0.05. **(B)** The paired-pulse facilitation was similar between Sarm1 knockdown and WT mice. The data represent the mean ± SEM. **(C)** The representative NMDAR (indicated by the gray line) and AMPAR (indicated by the black line) mediated currents from WT and Sarm1 knockdown CA1 neurons are shown in the upper panel. The ratio of NMDA to AMPA current is shown in the lower panel. The data indicate the mean plus SEM. *P < 0.05. The sample sizes (*n*) are indicated.

We then conducted a series of electrophysiology studies to corroborate the involvement of Sarm1 in synaptic plasticity. An extracellular field recording in Schaffer collateral-CA1 synapses was performed using acute hippocampal slices. We first examined the NMDAR-dependent LTP induced by TBS. Consistent with the higher calcium influx after glutamate stimulation and the higher dendritic spine density, the fEPSPs of Sarm1 knockdown hippocampal slices were enhanced by approximately 25% compared to the wild-type littermates (**Figure 3A**). It suggests that Sarm1 knockdown results in a hyper-LTP.

To dissect the origin of the disturbed NMDAR-dependent LTP, paired-pulse stimulation was used to test the ability of presynaptic terminals to release vesicles. The ratio of paired-pulse stimulation indicated that vesicle release was comparable between Sarm1 knockdown and wild type neurons (**Figure 3B**). It suggests that Sarm1 knockdown hippocampi have normal presynaptic function. We then analyzed the relative contribution of the NMDA and AMPA receptors to the EPSCs that were evoked by stimulation from the Schaffer collateral afferent. We found that the NMDA/AMPA ratio was significantly increased in Sarm1 knockdown neurons (**Figure 3C**). Taken together, these data indicate that reduced Sarm1 expression affects the synaptic response through a post-synaptic mechanism, which regulates the conductivity of NMDAR and AMPAR.

**FIGURE 4:**
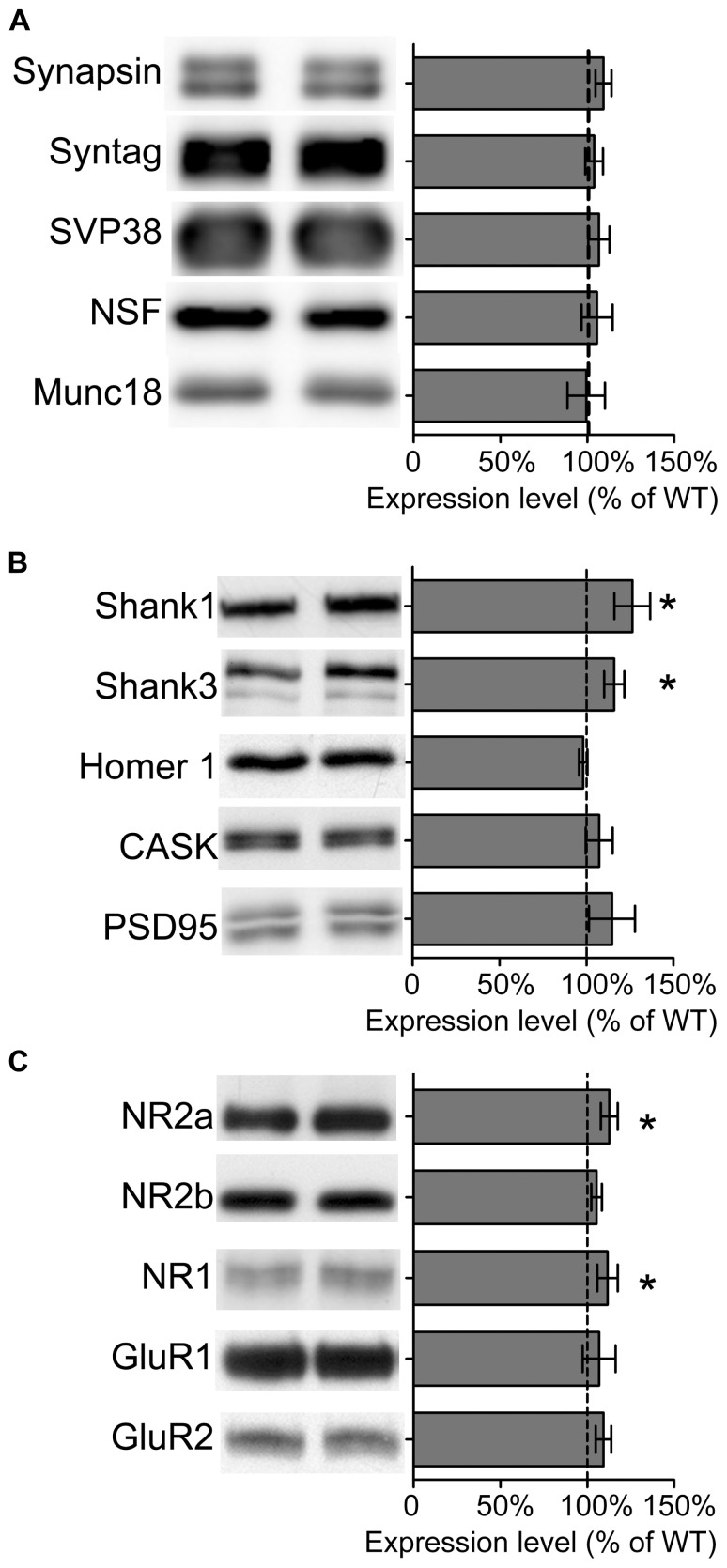
**Expression of some synaptic proteins are altered in Sarm1 knockdown mice. (A)** Presynaptic proteins. **(B)** Post-synaptic protein. **(C)** Glutamate receptors. The synaptosomal fractions of the hippocampi were analyzed by immunoblotting with antibodies, as indicated. Five animals for each genetic background were used for analysis. The data represent the mean ± SEM normalized to the WT levels. *P < 0.05.

We also analyzed the composition of synaptic proteins by isolating crude synaptosomal fractions (P2) from adult wild type and Sarm1 knockdown hippocampi. The results showed that the expression levels of presynaptic proteins, including synapsin, synaptotagmin, SVP38, NSF and Munc18, were not altered (**Figure 4A**), echoing the normal paired-pulse facilitation in Sarm1 knockdown brains. The only significant differences were the expression of post-synaptic scaffold proteins and NMDA receptor subunits, including Shank1, Shank3, NR1, and NR2a (**Figures 4B,C**). The increases were small (~12–25%) but significant. These changes of post-synaptic protein levels provide a potential molecular explanation for hyper-NMDAR-dependent LTP in Sarm1 knockdown mice.

### Sarm1 KNOCKDOWN ALSO RESULTS IN IMPAIRED mGluR-DEPENDENT LTD

**FIGURE 5:**
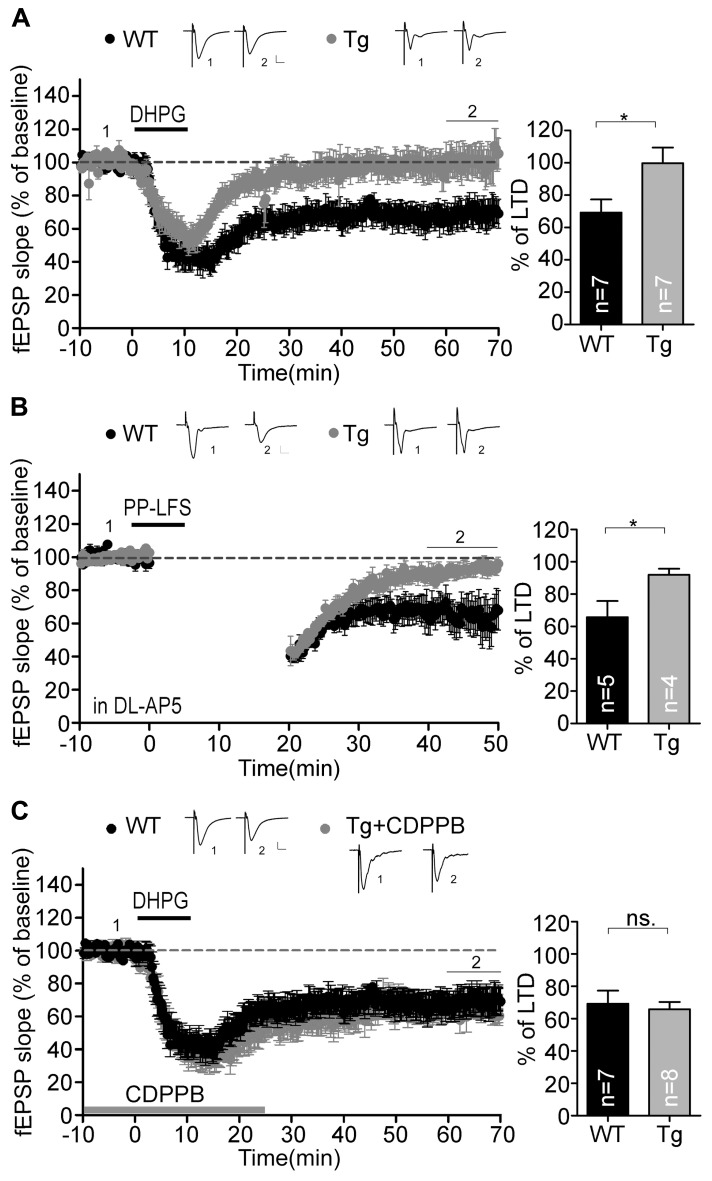
**Metabotropic GluR-dependent LTD is defective in the Sarm1 knockdown hippocampus.** Chemically and electrically induced LTD were recorded in the hippocampal CA1. The fEPSP is shown plotted as a function of time, and the representative traces at the indicated time points (labeled by 1 and 2 in the figure) are shown above. mGluR-dependent LTD induced by **(A)** DHPG and **(B)** paired-pulse low frequency stimulation was impaired in Sarm1 knockdown brains. **(C)** CDPPB was bath applied from the baseline recording for 25 min and showed profound rescue of DHPG-induced LTD in the Sarm1 knockdown hippocampus. **(A**–**C)** The horizontal dashed lines indicate the average value of the normalized amplitude during the control period. Calibration: 0.2 mV, 10 msec. For both LTP and LTD, histograms summarizing the average of the fEPSPs of the last 10 min of recording are shown in the right panels. The data represent the mean plus SEM (for histograms) or ± SEM (for fEPSP slopes). The sample sizes (*n*) are indicated. *P < 0.05; ns, not significant.

In addition to NMDAR-dependent LTP, we investigated LTD in the Sarm1 knockdown hippocampus. We were interested in the mGluR-dependent LTD because its involvement in synaptic pathophysiology has been confirm in two syndromic forms of autism with intellectual disability, tuberous sclerosis complex (*TSC1*^+^^/^^-^, TSC2^+^^/^^-^) and fragile X syndrome (*Fmr1*^-^^/^^-^; Bear et al., 2004; Zoghbi and Bear, 2012). Activation of mGluR triggers rapid local translation of proteins required for the endocytosis of AMPA receptors. This activity reduces the surface expression of AMPA receptors and leads to the formation of mGluR-induced LTD. Because Sarm1 has been shown to influence the ASK1-MKK-JNK/p38 pathway in neurons (Chuang and Bargmann, 2005; Chen et al., 2011), which is required for mGluR downstream signaling (Li et al., 2007; Moult et al., 2008), we asked whether Sarm1 could also participate in the synaptic activity that was regulated by mGluR. Two independent protocols were used to measure mGluR-LTD, including chemically and electrically induced LTD, either by treating with DHPG, an agonist of group I mGluR, or by paired pulse-low frequency stimulation (PP-LFS). The results of both experiments indicated that none of stimulations was able to induce LTD in Sarm1 knockdown brains, because compared to a wild-type control, Sarm1 knockdown brains exhibited a greatly reduced magnitude of DHPG- and PP-LFS-induced LTD in the hippocampus (**Figures 5A,B**). To confirm that the deficiency of mGluR signaling in Sarm1 knockdown mice, CDPPB, was added prior to the administration of DHPG. CDPPB functions as a positive allosteric modulator of mGluR5, which facilitates the effect of DHPG on mGluR. Thus, if the defect of Sarm1 knockdown neurons in response to DHPG is due to the deficit of mGluR signaling, the treatment of CDPPB is expected to rescue the defects in LTD (Auerbach et al., 2011; Verpelli et al., 2011). Indeed, enhancing mGluR5 signaling by CDPPB reversed the LTD defect in Sarm1 knockdown (**Figure 5C**). It confirmed that the deficits in mGluR5 signaling were involved in the LTD impairment of Sarm1 knockdown mice.

### CDPPB TREATMENT AMELIORATES THE BEHAVIORAL DEFECTS IN Sarm1 KNOCKDOWN MICE

**FIGURE 6:**
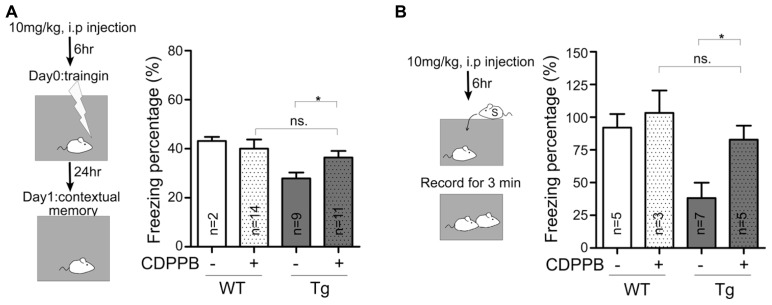
**Enhancement of mGluR5 signaling ameliorates the behavioral defects in Sarm1 knockdown mice.** CDPPB (10 mg/kg of animal body weight) or a vehicle control was intraperitonally injected into mice six hours before the behavioral assay. **(A)** The results of contextual fear conditioning are shown. **(B)** The results of reciprocal social interaction are shown. CDPPB did not obviously influence the behaviors of WT littermates. However, CDPPB rescued the defects observed in Sarm1 knockdown mice. The sample sizes (*n*) are indicated in the column. *P < 0.05; ns, not significant.

A recent study that focused on fragile X and tuberous sclerosis mouse models suggests that there is shared synaptic pathology in mGluR signaling (Auerbach et al., 2011). In particular, *TSC2*^+^^/^^-^ mutant mice display reduced mGluR-LTD responses, which are associated with intellectual disabilities, and these symptoms can be improved by enhancing mGluR signaling (Auerbach et al., 2011). Because Sarm1 knockdown mice are characterized by autistic phenotypes (Lin and Hsueh, 2014) and because the data above showed an impairment of mGluR-dependent LTD in Sarm1 knockdown mice, we asked whether a pharmacological treatment could reverse the defects in Sarm1 knockdown mice, specifically dysregulated mGluR-dependent LTD and behavioral defects. Reciprocal social interaction and contextual fear conditioning were examined. We have previously demonstrated that Sarm1 knockdown affects these two behavior paradigms in mice (Lin and Hsueh, 2014). CDPPB was injected i.p. into Sarm1 knockdown and WT littermate mice prior to the behavioral assays (**Figures 6A,B**). Consistent with the effects of CDPPB on electrophysiology (**Figure 4C**), we found that the systemic administration of CDPPB also ameliorated the behavioral defects observed in Sarm1 knockdown mice during contextual fear conditioning (**Figure 6A**) and reciprocal social interaction (**Figure 6B**). These results strengthen the link between mGluR-dependent LTD and Sarm1-regulated neuronal function, and they provide a possible treatment to ameliorate the behavioral defects caused by Sarm1 deficiency.

## DISCUSSION

In this report, we demonstrate that Sarm1 knockdown causes synaptic dysfunction, including increased Ca^2^^+^ influx upon glutamate treatment *in vitro* as well as enhanced NMDAR-dependent LTP and impaired mGluR-dependent LTD in hippocampi. The behavioral rescue by CDPPB, a positive allosteric modulator of mGluR, further supports a role for mGluR signaling in the pathogenesis of autistic-like behaviors that are caused by Sarm1 knockdown. Metobotrophic GluR signaling activity is known to mediate spine shrinkage and degradation (Oh et al., 2013; Ramiro-Cortes and Israely, 2013), which may account for the increased spine density that was observed in the CA1 pyramidal neurons. Our data also suggest that there are changes in post-synaptic protein composition, including NR1, NR2a, and Shank proteins, in Sarm1 knockdown brains. These synaptic proteins not only directly contribute to synaptic responses but are also known to be involved in the etiology of autism spectrum disorder (ASD). For example, all of the members of the Shank protein family that indirectly link NMDAR and mGluR were identified as autism causative genes (Durand et al., 2007; Hung et al., 2008; Berkel et al., 2010; Pinto et al., 2010; Peca et al., 2011; Sato et al., 2012; Schmeisser et al., 2012; Won et al., 2012). Mutations in the NR2b gene were consistently identified in patients with autism (O’Roak et al., 2012). These observations support the current hypothesis that the intellectual disabilities associated with autism are caused by unbalanced synaptic activity. Both overly strong or overly weak synaptic function can harm neural plasticity and results in autistic-like behaviors and impaired intellectual performance (Auerbach et al., 2011; Sui and Chen, 2012; Zoghbi and Bear, 2012; Lin et al., 2013; Nuytens et al., 2013). However, the detailed mechanisms underlying the role of Sarm1 in LTD and LTP have yet to be determined. For example, it will be intriguing to further elucidate whether Sarm1 regulates mGluR and NMDAR signaling through independent mechanisms or primarily through an effect on the mGluR pathway that modulates the NMDAR response indirectly. Because Shank proteins were upregulated in Sarm1 knockdown neurons and because Shanks link NMDAR to mGluR, Sarm1 knockdown may also influence both NMDAR and mGluR via Shanks.

Based on previous studies, Sarm1 likely regulates synaptic plasticity through both direct and indirect mechanisms. Our previous study showed that Sarm1 is widely distributed in different neuronal subcellular regions, including in the synapses (Chen et al., 2011). Sarm1 puncta were partially co-localized with PSD-95 (Chen et al., 2011). The synaptic localization of Sarm1 can be maintained by direct interaction with syndecan-2, a transmembrane heparan sulfate proteoglycan that is highly enriched at the synapse. Synaptic Sarm1 may recruit signaling molecules, such as the components of the MKK4-JNK pathway, to synapses. In *C. elegans*, the synaptic localization of Tir-1, a Sarm1 homolog, is determined by the interaction between its SAM domain and UNC-43 (CaMKII homolog; Chuang and Bargmann, 2005). In mammalian neurons, CaMKII is also a key component in synaptic plasticity, and it is concentrated at the post-synaptic region (Lisman et al., 2002; Lee et al., 2009). It seems likely that Sarm1 also interacts directly with CaMKII to control synaptic plasticity.

Sarm1 also regulates neuronal morphogenesis (Chen et al., 2011) and the expression of inflammatory cytokines in the brain (Lin et al., 2014). In addition to dendritic arborization and axonal differentiation, we showed here that Sarm1 knockdown can also influence dendritic spine density. Because proper neuronal morphology is essential for effective communication between neurons, the abnormalities of neuronal morphology caused by Sarm1 knockdown may result in dysregulated circuits between hippocampal CA1 and CA3, thus impairing synaptic plasticity in Sarm1 knockdown mice. On the other hand, Sarm1 knockdown causes dysregulated cytokine expression in the brain, including changes in IL-1β, IL-12, CCL5, TNF-α ,and IFN-β production (Lin et al., 2014). IL-1ß and TNF-α are known to regulate synaptic plasticity (Del Rey et al., 2013; Gruber-Schoffnegger et al., 2013). Thus, it is also likely that inflammatory cytokines mediate Sarm1 effects on synaptic plasticity.

In the rescue experiments, CDPPB treatment was able to ameliorate the behavioral defects found in the Sarm1 knockdown mice, suggesting a critical role for mGluR signaling in the Sarm1 pathway. Because of the function of Sarm1 in innate immune responses, our data also reveals a potential treatment for inflammation-related neurological disorders by using mGluR agonists.

## AUTHOR CONTRIBUTIONS

Chia-Wen Lin designed and carried out the experiments, including calcium imaging, immunoblots, electrophysiological recording and behavioral analysis, and drafted the manuscript. Chiung-Ya Chen designed and carried out the Golgi stain and drafted the manuscript. Sin-Jhong Cheng designed and carried out the electrophysiological recordings and drafted the manuscript. Hsiao-Tang Hu designed and carried out the calcium imaging experiment shown in **Figure 1E**. Yi-Ping Hsueh designed the experiments and drafted the manuscript. All of authors read and approved the final version of manuscript.

## Conflict of Interest Statement

The authors declare that the research was conducted in the absence of any commercial or financial relationships that could be construed as a potential conflict of interest.
